# SH2 Domain Binding: Diverse FLVRs of Partnership

**DOI:** 10.3389/fendo.2020.575220

**Published:** 2020-09-18

**Authors:** Rachel Jaber Chehayeb, Titus J. Boggon

**Affiliations:** ^1^Yale College, New Haven, CT, United States; ^2^Department of Molecular Biophysics and Biochemistry, New Haven, CT, United States; ^3^Department of Pharmacology, New Haven, CT, United States; ^4^Yale Cancer Center, Yale University, New Haven, CT, United States

**Keywords:** Src-homology 2, phosphotyrosine, FLVR motif, protein-protein interaction, signal transduction, protein domains

## Abstract

The Src homology 2 (SH2) domain has a special role as one of the cornerstone examples of a “modular” domain. The interactions of this domain are very well-conserved, and have long been described as a bidentate, or “two-pronged plug” interaction between the domain and a phosphotyrosine (pTyr) peptide. Recent work has, however, highlighted unusual features of the SH2 domain that illustrate a greater diversity than was previously appreciated. In this review we discuss some of the novel and unusual characteristics across the SH2 family, including unusual peptide binding pockets, multiple pTyr recognition sites, recognition sites for unphosphorylated peptides, and recently identified variability in the conserved FLVR motif.

## Introduction

In 1986, a new domain was identified within v-*src*, the transforming gene of the Rous sarcoma virus (1–3). This region had high sequence similarity across the then-known cytoplasmic protein-tyrosine kinases, and a conserved location N-terminal to the kinase domain (the first homology region identified in Src). The domain was termed the Src homology 2 (SH2) domain, and its identification heralded a new era in the understanding of molecular interactions and cellular signaling (2, 4). SH2 domains have been critical for development of key concepts such as the dependency of cytosolic signaling on post-translational modification-regulated protein interactions (5–8), and the modularity of protein domains (3, 9, 10). Over the years, extremely well conserved molecular mechanisms have been revealed which are used by SH2 domains to mediate their effects (11–13). These canonical features are well-documented, but unusual features also occur and increase the diversity of the fold. In this review, we discuss these unusual features and how they exhibit divergence from canonical SH2 domain architecture.

## Global Features of The Canonical SH2 Domain

The primary molecular role of the SH2 domain is to directly bind phosphotyrosine (pTyr) residues (14). This is central to propagation of signaling by receptor and non-receptor tyrosine kinases such as the insulin receptor and the JAK kinases, so SH2 domains are critical to a range of fields including endocrinology (5, 15–18). The SH2-pTyr interaction is broadly independent of folding of the pTyr-ligand, and can be observed for denatured Tyr peptides (19–22), but is distinct from recognition of pTyr by for example the phosphotyrosine binding (PTB) domains (5). Thus, the binding of SH2 domains to short linear peptide motifs can be predictive for the interactome of specific SH2 domains (20–23). SH2 interaction selectivity has yielded extensive knowledge of their binding partner preferences and signaling networks (2, 5).

**Figure 1:**
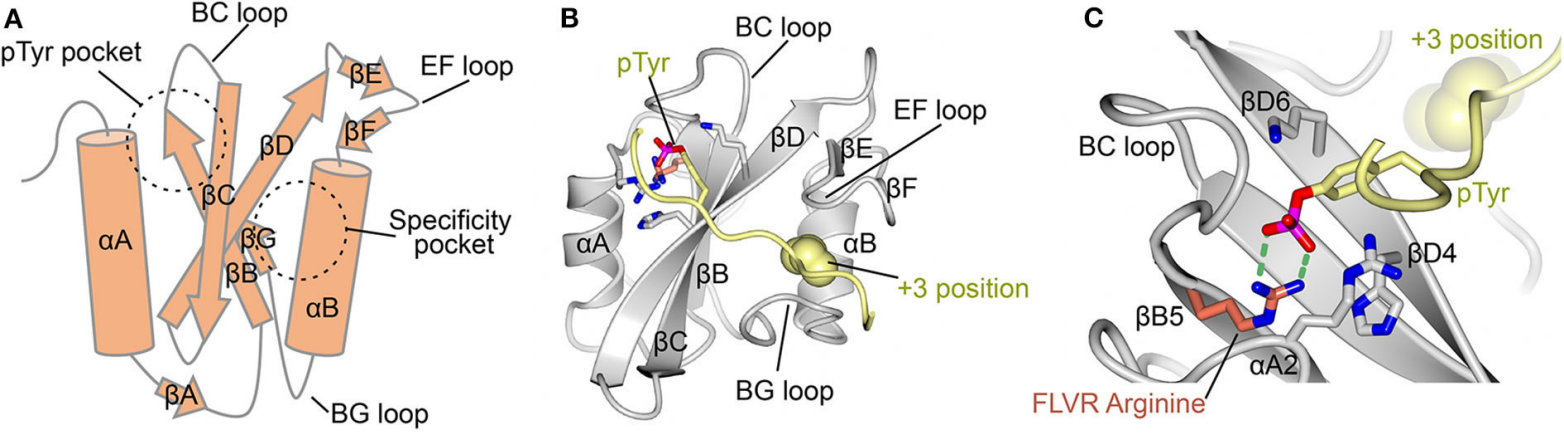
Canonical SH2 domain binding to phosphotyrosine peptide. **(A)** Schematic diagram of the SH2 domain. Important secondary structure elements are indicated. Locations of the pTyr binding pocket and the specificity pocket are shown. **(B)** Crystal structure of the LCK SH2 domain in complex with a pTyr peptide (PDB ID: 1LCJ) (28). SH2 domain shown in gray and the pTyr peptide in yellow. pTyr and important residues for its binding are shown in stick format. Ile three residues C-terminal to pTyr (+3 position) is shown in space filling format. **(C)** Close up of the pTyr binding pocket of LCK showing the FLVR arginine at position βB5 in orange and illustrating its H-bonding to pTyr (green dashed lines). Other important residues for pTyr binding include those at positions αA2, βD4, and βD6.

The mechanisms for SH2-ligand interaction are well-defined, with the first cohort of structures for this ~100 amino acid fold determined in 1992 and 1993 (24–28). These structures showed that the SH2 domain consists of a central β-sheet flanked by two α-helices. They revealed that the phosphorylated peptide binds perpendicularly to the β-sheet and docks into two abutting recognition sites formed by the β-sheet with each of the α-helices. This bidentate, or “two-pronged plug” (26), interaction provides both a deep basic pTyr binding site, and a specificity pocket that usually recognizes an amino acid three residues C-terminal to the pTyr (termed the +3 position), a mode of interaction that is consistent *in vitro* and in cells (3, 21, 22, 29) (Figure 1A). The nomenclature for the fold defines the antiparallel β-strands as βA-βG and the helices as αA and αB, with loops named by the flanking secondary structure (Figure 1B) (28). Thus, the pTyr pocket is canonically defined by residues of αA, βB, βC, βD, and by the BC “phosphate binding loop” (Figure 1C); and the specificity pocket by residues of αB, βG, and the BG and EF loops.

Evolutionarily, the SH2 domains appear early in the eukaryotic phylogenetic tree and are thought to have co-evolved with tyrosine kinases to the complex array of pTyr-responsive signaling is found in humans (30, 31). Indeed, an ancestral SH2 domain appears to have been identified in SPT6, a transcription elongation factor universally present from yeast to humans. As discussed below, SPT6 maintains the overall SH2 fold but binds to phospho-serine and phospho-threonine thus providing a stepping stone to pTyr binding (31, 32). Unusual SH2 domains have also been acquired and evolved by some bacteria, presumably for invasive purposes, and below we discuss some of those contained in the *Legionella* genome (33, 34).

## Arginine βB5 (The FLVR of an SH2 Domain) and Other Conserved Features

Within the canonically defined pTyr pocket there are conserved residue motifs (21). The most critical motif for pTyr binding includes an arginine at the fifth position of βB, βB5, as part of a highly conserved “FLVR” or “FLVRES” amino acid motif (12, 25) (Figure 1C). As much as half of the free energy of binding is lost on point mutation of this residue (35, 36) resulting in a 1,000-fold reduction in binding affinity (25, 26), and it provides a floor at the base of the deep pTyr pocket which allows specificity toward pTyr over pSer/pThr (4, 8). The FLVR arginine residue is considered key to pTyr recognition, and is conserved in all but 3 of the 120+ human SH2 domains (31).

Other conserved residues which often work in concert with βB5 to coordinate pTyr have been identified. The most prominent are basic residues (arginine or lysine) at positions αA2 and βD6 (Figure 1C). Coordination of pTyr by both αA2 and βD6 is rarely observed in the same SH2 domain and this observation has allowed the definition of two major classes, the Src-like (with a basic residue at αA2), and the SAP-like (with a basic residue at βD6) SH2 domains, referencing two of the most well studied members of the family (33). Experimentally, however, Arg βB5 is the residue most often targeted by point mutagenesis to interrupt SH2-pTyr binding (37, 38).

Outside of the canonical binding site, which provides recognition of the pTyr and +3 positions, interfaces have been found to contribute to binding at a range extending to the −6 and +6 positions (7, 21). Larger interaction and alternative surfaces have also been observed, for example to achieve high FGFR1 selectivity the N-terminal SH2 domain of PLCγ1 uses an extended surface, but its C-terminal SH2 domain does not and is consequently a weaker binder (39). An alternate surface is also used by the SAP SH2 domain which interacts with the SH3 domain of Fyn using a region distal to its pTyr binding site (40). Despite these findings, however, the foundational conserved mode of binding for most SH2 domains to pTyr ligands is centered on the interactions of pTyr and the +3 position. Nonetheless, over the course of study of SH2 domains exceptions to these general rules have been observed.

## Unusual SH2 Domains

The exceptions to the canonical “two-pronged plug” binding observed thus far create a diversity in the recognition patterns by which SH2 domains can bind their partners. These exceptions include unusual binding pockets, unique specificities, and dependency of oligomeric state for binding. Below, we highlight some of the mechanisms SH2 domains use to select binding partners and discuss diversity within the pTyr binding site, starting with ancestral SH2 domains before proceeding to eukaryotic SH2 domains.

### Ancestral and Borrowed SH2 Domains

**Figure 2:**
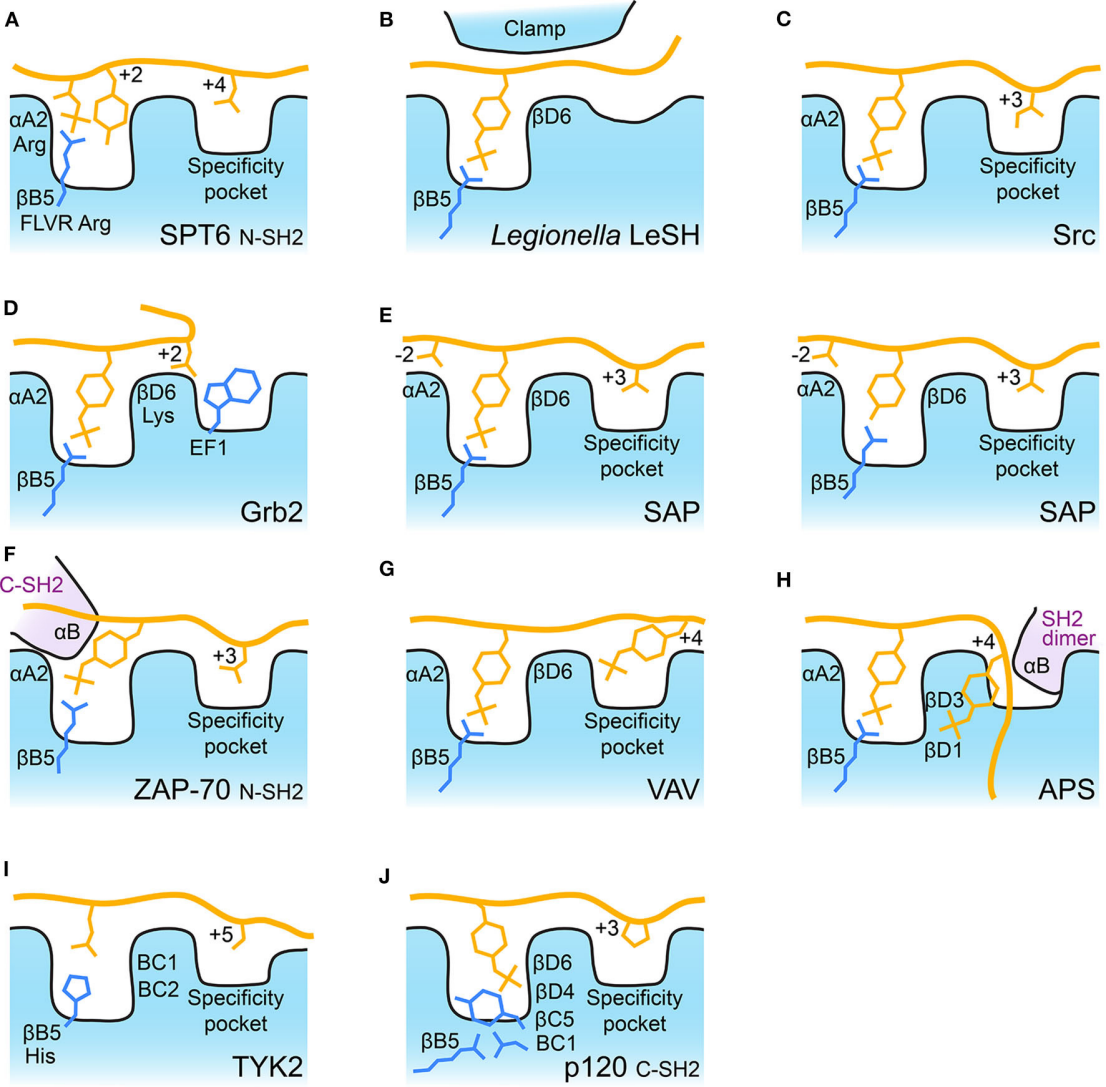
Schematic of SH2 binding modes. Schematic illustration of some of the major SH2 binding features discussed. For each panel the FLVR arginine residue, βB5, is indicated and depicted. Bound peptide is shown with some residues important for selectivity indicated. The FLVR Arg is shown in each schematic. Position αA2 is often Arg and βD6 is often Lys. **(A)** SPT6 bound to pThr peptide. **(B)** *Legionella* LeSH clamping pTyr peptide. βD6 is Arg in LeSH. **(C)** Src bound to pTyr peptide. **(D)** Grb2 illustrating Trp blockage of the canonical specificity pocket. **(E)** SAP bound to phosphorylated and unphosphorylated peptides. **(F)** ZAP-70 N-SH2 domain illustrating the contribution of residues from its C-SH2 αB helix to pTyr binding. **(G)** VAV bound to doubly phosphorylated peptide. **(H)** APS bound to doubly phosphorylated peptide with αB helix from its dimer partner indicated, βD1 and βD3 are both Lys residues. **(I)** TYK2 bound to cytokine receptor tail. The FLVR residue is a His (indicated), BC1 is Ser and BC2 is Thr. **(J)** p120RasGAP (p120) C-SH2 bound to phosphorylated peptide. The BC1 residue is an Asp which makes a salt bridge to the FLVR Arg, βB5. βC5 is a Tyr which blocks pTyr interaction with Arg, βB5. βD4 is Arg and βD6 is Lys, both of which coordinate pTyr.

Probably the most ancient SH2 domain discovered to date is found in SPT6, an essential transcription elongation protein. This protein contains tandem SH2 domains which are the only two SH2 domains in yeast. They pack against one another and recognize extended phosphorylated serine and threonine peptides of RNA polymerase II (41). The C-terminal SH2 domain lacks a canonical phospho-binding site (32, 42), but instead has a pocket on its back side which binds a pSer in its binding partner. In contrast, the N-terminal SH2 domain has a near canonical phospho-binding pocket which recognizes pThr, and its recent structure-guided analysis showed that the N-SH2 pocket preferentially binds pThr followed by Tyr (41). This pT-X-Y motif makes use of the FLVR arginine to coordinate the pThr's phosphate, but the Tyr is also oriented into this pocket in a manner similar to the aromatic region of a canonical pTyr-SH2 interaction. The coordination of both pThr and Tyr by SPT6 therefore resembles a canonical pTyr-SH2 interaction, making this potentially the evolutionary stepping-stone to SH2-mediated pTyr recognition (41) (Figure 2A).

In Gram-negative bacteria, a large number of SH2 domains have been discovered in the *Legionella pneumophila* genome (33). These are probably the result of horizontal gene transfer but expand the SH2 group to prokaryotes. The *Legionella* SH2 domains bind using a conserved FLVR arginine and coordinate pTyr in the canonical fashion, but show minimal selectivity for residues in the +3 region due to the lack of a specificity pocket. The structure of the LeSH SH2 domain demonstrates this novel architecture, whereby a large insert (located in the same region as the EF loop) is thought to undergo a “clamping” conformational reorganization which grasps the pTyr peptide and facilitates high affinity binding with low sequence selectivity (33) (Figure 2B). This is a completely divergent mechanism of achieving tight SH2-pTyr binding compared to all other SH2 domains, so it is interesting to speculate on the potential role of these newly identified SH2 domains in hijacking host cell signaling cascades.

### Drivers of Substrate Selectivity

Among eukaryotes there is also great diversity in SH2 selectivity. This is most often driven by the selectivity pocket, and much work has been conducted to delineate the ability of individual SH2 domains to recognize specific sequences particularly at the pTyr +3 position (21, 29, 43), and to understand the structural and molecular basis for these selectivities (26, 28, 44) (Figure 2C). These studies have found that for some SH2 domains the mode of peptide binding can be variable. For example, in PLCγ1 a number of pTyr peptides have been shown to bind the FLVR arginine residue but to display significant differences in peptide binding (45). In contrast, the adaptor protein Grb2 and its homolog Gads demonstrate high selectivity by using canonical FLVR arginine interactions to recognize pTyr but an altered selectivity pocket, where a Trp residue contributed by the EF loop forces the bound peptide to turn, to achieve a strong preference for Asn at the +2 position (46–48) (Figure 2D). Furthermore, post-translational modifications can alter peptide selectivity, and this has been observed for multiple members of the Src-family kinase SH2 domains where phosphorylation (for example in Lck at residue Tyr192) alters the selectivity preference at the +2/+3 position (49–51).

Not all SH2 domains are restricted to binding pTyr. For example, the SH2 domain of SAP is mutated in X-linked lymphoproliferative syndrome (XLP) and can bind both phosphorylated and non-phosphorylated tyrosine peptides of the lymphocyte receptor SLAM (52, 53). This ability to bind unphosphorylated Tyr peptides is highly unusual for SH2 domains and is achieved by combining FLVR arginine interactions with either pTyr or Tyr and an extended “three-pronged” interaction (Figure 2E). SAP uses the pTyr and the +3 hydrophobic pocket to bind SLAM, but also utilizes a pocket which recognizes positions N-terminal to the pTyr (52). The extended interaction allows SAP to bind phosphorylated as well as unphosphorylated peptides, albeit with ~2–5 fold lower affinity than binding to the phosphorylated peptide (52, 53).

### Binding Dual pTyrs by Tandem SH2 Domains

Some SH2 domains can bind peptides which contain multiple pTyr residues. These include the non-receptor tyrosine kinase ZAP-70, and its homolog, Syk that contain tandem SH2 domains (54, 55) which form a tight module and are linked by a small interdomain coiled coil domain (56). Their role is two-fold; they are critical for autoregulation of catalytic activity by orienting the interdomain coiled coil to pack against the kinase domain, and they recruit ZAP-70 to doubly-tyrosine phosphorylated ITAM receptors. To bind to ITAM cytoplasmic tails, the C-terminal ZAP-70 SH2 domain uses its conserved FLVR arginine and a canonical “two-pronged plug” type interaction (56). In contrast, the N-terminal SH2 domain uses a modified canonical interaction, where the FLVR arginine coordinates pTyr, but the binding site lies at the interface of the two domains and is completed by residues of the C-terminal SH2 domain so that recognition of the second pTyr requires both SH2 domains (Figure 2F) (56). This intercalated pTyr binding site is functionally important for ZAP-70 because its engagement in the context of ITAM binding induces a significant conformational change which releases autoinhibition and is an important step toward full kinase activity (54, 55, 57).

### Binding Dual pTyrs by The Same SH2 Domain

Syk is itself phosphorylated, and two of these phosphosites are closely spaced (Y342 and Y346). Recognition of this tandem phosphorylated region is achieved by the SH2 domains of VAV and PLCγ1 (58, 59) in a phospho-state dependent fashion (60). The primary pTyr binding site is canonical and uses the FLVR arginine of VAV/PLCγ1, however, a second pTyr binding site is formed by basic residues of the βD strand and BG loop (Figure 2G). Interestingly, binding of the dual phosphopeptide by PLCγ1 SH2 induces significant conformational changes in the domain compared to when it is bound to mono-phosphorylated peptides (59). Src has also been shown to be able to bind to dual phosphorylated peptides, and structural and thermodynamic analysis of its binding to a doubly phosphorylated PDGF receptor peptide illustrate the coordination of a second pTyr by a basic residue of the βD strand as a potentially more common mechanism for SH2 domains (61).

### Binding Quadruple pTyrs by an SH2 Module

Dual phosphotyrosine binding facilitated by βD residues is further expanded on by the adaptor protein APS, a substrate of insulin receptor tyrosine kinase which binds directly to the phosphorylated “activation loop” of the kinase (62). The SH2 domain of this adaptor protein exists as a dimer mediated primarily by its long αB helix (62). In the dimer, both extended αB helices reciprocally interrupt the canonical specificity pocket site of the other SH2 domain, so APS has reduced ability to bind to canonical extended backbone pTyr substrates in the normal fashion (62, 63). Instead, APS binds to two pTyr residues of insulin receptor with a two-pronged site that uses the canonical FLVR arginine pocket and a basic patch created by Lys residues on strand βD (Figure 2H). This is a dual pTyr recognition by both copies of the APS dimer that creates a quadruple phosphotyrosine binding module. From a signaling point of view, the quadruple phosphotyrosine binding does not interrupt insulin receptor's kinase activity, instead it facilitates phosphorylation of the APS C-terminal tail and recruitment of further signaling molecules to the insulin receptor (15).

## A Diversity of FLVR

In the above examples, selectivity is mostly driven by alterations in the selectivity pocket or by unusual selectivity sites. These interactions are, however, extremely similar in their utilization of the FLVR arginine to coordinate pTyr (or pThr in SPT6 and Tyr in SAP). In contrast, there are very few SH2 domains which are not conserved at the FLVR arginine and consequently cannot bind pTyr *via* this residue. In humans these are RIN2, TYK2, and SH2D5; the FLVR arginine is replaced by histidine in RIN2 and TYK2, and a tryptophan in SH2D5. The consequences of this difference have been shown structurally for TYK2, a non-receptor tyrosine kinase of the Janus kinase (JAK) family (64). For all four JAKs (JAK1, JAK2, JAK3, and TYK2), the SH2 domain is a subsidiary component of a larger 4-sub-domain receptor binding module where the SH2 domains mediate a portion, but not the entirety, of the JAK-cytokine receptor interaction (64–67). For receptor binding, the SH2 domains use their canonical peptide substrate binding cleft but do not maintain the requirement to bind pTyr. They often bind an acidic Glu residue instead of pTyr and use an extended specificity pocket to further recognize the receptor tail (68). Evolutionarily, this allowed the FLVR arginine to diverge, and in TYK2 it is a His residue (Figure 2I). The Janus kinases therefore represent a class of SH2 domain that mediates protein-protein interactions without requiring FLVR arginine-pTyr recognition.

Our recent discovery has further illustrated the diversity of SH2 domains. One of the first SH2 proteins to be identified was p120RasGAP (RasGAP, RASA1) (69–71), and for 30 years it was thought to contain two canonical SH2 domains (termed the N- and C-terminals SH2s) (72–74). We investigated p120RasGAP, and our crystal structure and mutagenic analysis of the N-terminal SH2 domain showed this to be correct for the N-terminal domain (75), however, we found that the C-terminal SH2 domain assumes a completely unexpected mode of pTyr recognition (76). Instead of contacting the conserved phosphotyrosine as observed in every other SH2-pTyr interaction, the FLVR arginine at residue position 377 makes a salt bridge to an aspartic acid at position 380. This salt bridge is unprecedented in SH2 domains and requires that pTyr binding is achieved by a unique mechanism that uses multi-dentate recognition by basic residues at βD4 and βD6, and by residues in the BC loop (Figure 2J). The reason for this unusual binding mode is currently unknown, but it is highly conserved over evolution, only diverging in extremely ancient examples of p120RasGAP (76). Despite being one of the first identified and best studied SH2 domain proteins, the C-terminal SH2 domain of p120RasGAP has revealed another mechanism by which SH2 domains can achieve their purpose as protein interaction domains.

## Conclusions

The SH2 domain is one of the best studied and understood protein-interaction folds. It has a wide range of functions, and its interactions with partner proteins are generally well-understood. Although the key findings made in the early 1990's describing the molecular basis of SH2-pTyr interactions have been shown to be remarkably robust across the fold and across evolution, there are exceptions to these findings. These include unusual peptide binding pockets, extra pTyr interaction sites, recognition and binding of unphosphorylated peptides, and unusual pTyr recognition sites. The recent discovery of a unique pTyr binding site in p120RasGAP, one of the earliest identified SH2 domains, illustrate the continued discoveries of novelty and diversity among this important group, and the surprises it continues to yield.

## Author Contributions

All authors listed have made a substantial, direct and intellectual contribution to the work, and approved it for publication.

## Conflict of Interest

The authors declare that the research was conducted in the absence of any commercial or financial relationships that could be construed as a potential conflict of interest.

## Abbreviations

APS: Adapter protein with pleckstrin homology and Src homology 2 domains
FGFR1: Fibroblast growth factor receptor 1
Gads: Grb2-related adaptor downstream of Shc
Grb2: Growth factor receptor-bound protein 2
ITAM: Immunoreceptor tyrosine-based activation motif
JAK: Janus kinase
LeSH: *Legionella* SH2
PDGF: Platelet-derived growth factor
PLCγ1: 1-phosphatidylinositol 4,5-bisphosphate phosphodiesterase gamma-1
PTB: phosphotyrosine binding domain
pTyr: Phosphotyrosine
RIN2: Ras and Rab interactor 2
SAP: SLAM-associated protein
SH2: Src homology 2
SH2D5: SH2 domain-containing protein 5
SLAM: Signaling lymphocytic activation molecule
SPT6: Transcription elongation factor SPT6
Syk: Spleen tyrosine kinase
TYK2: Non-receptor tyrosine-protein kinase TYK2
XLP: X-linked lymphoproliferative syndrome
ZAP-70: ζ-chain associated protein of 70kDa.
